# Computational Modeling of Tumor Response to Drug Release from Vasculature-Bound Nanoparticles

**DOI:** 10.1371/journal.pone.0144888

**Published:** 2015-12-14

**Authors:** Louis T. Curtis, Min Wu, John Lowengrub, Paolo Decuzzi, Hermann B. Frieboes

**Affiliations:** 1 Department of Bioengineering, University of Louisville, Louisville, Kentucky, United States of America; 2 Department of Engineering Sciences and Applied Mathematics, Northwestern University, Chicago, Illinois, United States of America; 3 Department of Mathematics, University of California, Irvine, California, United States of America; 4 Department of Biomedical Engineering, University of California, Irvine, California, United States of America; 5 Chao Family Comprehensive Cancer Center, University of California, Irvine, California, United States of America; 6 Department of Translational Imaging and Department of Nanomedicine, Houston Methodist Research Institute, Houston, Texas, United States of America; 7 James Graham Brown Cancer Center, University of Louisville, Louisville, Kentucky, United States of America; Case Western Reserve University, UNITED STATES

## Abstract

Systemically injected nanoparticle (NPs) targeting tumor vasculature offer a venue for anti-angiogenic therapies as well as cancer detection and imaging. Clinical application has been limited, however, due to the challenge of elucidating the complex interplay of nanotechnology, drug, and tumor parameters. A critical factor representing the likelihood of endothelial adhesion is the NP vascular affinity, a function of vascular receptor expression and NP size and surface-bound ligand density. We propose a theoretical framework to simulate the tumor response to vasculature-bound drug-loaded NPs and examine the interplay between NP distribution and accumulation as a function of NP vascular affinity, size, and drug loading and release characteristics. The results show that uniform spatial distribution coupled with high vascular affinity is achievable for smaller NPs but not for larger sizes. Consequently, small (100 nm) NPs with high vascular affinity are predicted to be more effective than larger (1000 nm) NPs with similar affinity, even though small NPs have lower drug loading and local drug release compared to the larger NPs. Medium vascular affinity coupled with medium or larger sized NPs is also effective due to a more uniform distribution with higher drug loading and release. Low vascular affinity hampered treatment efficacy regardless of NP size, with larger NPs additionally impeded by heterogeneous distribution and drug release. The results further show that increased drug diffusivity mainly benefits heterogeneously distributed NPs, and would negatively affect efficacy otherwise due to increased wash-out. This model system enables evaluation of efficacy for vascular-targeted drug-loaded NPs as a function of critical NP, drug, and tumor parameters.

## Introduction

It is well known that chemotherapy success is hindered by cancerous tissue not following normal biological development. In addition to intrinsic and acquired cellular resistance thwarting drug response, heterogeneous blood supply, elevated interstitial pressure, and larger-than-normal inter-vascular distances in solid tumors prevent systemically-administered drug as well as nano-based therapeutics from attaining homogeneous distribution in sufficient concentrations. Uptake by the reticulo-endothelial system (RES) may further prevent adequate nanotherapeutic concentration at the tumor site. Even if drug were to be available in cytotoxic concentrations, quiescent cells in tumor hypoxic regions would remain largely unresponsive to cell-cycle dependent chemotherapeutics. These cells would regain access to adequate oxygen and nutrients once the overall population was thinned out by therapy, thus resuming the tumor growth.

By targeting tumors more effectively and reducing toxic side effects, nanotechnology-based therapy offers the possibility to improve cancer treatment efficacy [1–3]. In particular, encapsulation of chemotherapeutic drugs into liposomal molecules is an active research area. These nanoparticles offer potential benefits over chemotherapeutic agents alone, including increased drug bioavailability, decreased drug degradation and inactivation, as well as decreased off-site toxicity [4, 5]. Nevertheless, very few liposomal or other types of formulations have successfully crossed from the laboratory to clinical use. A major reason is that the combination of nanotechnology, drug, and tumor parameters is too complex for solely empirical evaluation. Mathematical and computational approaches have recently been developed as complementary tools to assist in this endeavor [6–19].

NPs can be targeted to tumor cells through variation in surface properties, shape and size. In particular, passive targeting for typically spherical NPs with diameters ranging between ∼ 50 and 300 nm can be achieved via the Enhanced Permeability and Retention (EPR) effect [20–22], which takes advantage of the typically leaky and fenestrated tumor vasculature. Variation in vascular structure due to stage, location, and type of cancer, however, hinder optimal performance [22, 23]. In contrast, vascular targeting aims to localize NPs onto vascular endothelial cells within the tumor vasculature by attaching ligands for endothelial cell receptors on the NP surface, and without necessarily exiting the vessels and interacting with the cancer cells themselves [24, 25]. Vascular targeting enables NPs to be larger than those targeted to tumor cells via the EPR effect, and thus carry more drug that can be released over longer periods of time. The drug could target tumor cells as well as the endothelial cells themselves (anti-angiogenic therapy) [26]. It has been shown that this strategy could be especially effective in targeting metastatic lesions [27]. Alternatively, the vascular-targeted NPs could carry agents allowing for detection or visualization of tumor vascular and tissue structures (e.g., [28]).

A numerical study evaluating NP distribution along a branched vessel for different NP sizes, shapes, and shear rates was presented in [29], finding that the binding rate is higher for NPs of smaller sizes and at lower shear rates. The vascular deposition of spherical NPs with sizes ranging between 700 nm and 3 μm was previously shown to decrease monotonically with NP diameter [30]. Further, in agreement with earlier results [31], it was shown that the strength of adhesion under flow decreases as the NP diameter increases [30]. A steady decrease in strength of adhesion for spherical particles larger than about 500 nm in diameter subjected to capillary flow has also been demonstrated theoretically [32]. We previously showed that NP surface properties, size, and shape could be tuned via mathematical modeling and computational simulation to enhance specific vascular target recognition, yielding firm and stable adhesion to endothelial cells experiencing flow-induced shear stress [17, 32, 33]. We further integrated a mesoscale representation of blood-borne NPs adhering to vascular endothelial cells with a model of vascularized tumor growth to evaluate the fraction of injected NPs accumulating within tumor vasculature as well as their spatial distribution as a function NP size and degree of vascular affinity for surface-bound ligands by [13]. The results showed an optimal vascular affinity could be identified providing for proper balance between NP uniform spatial distribution and accumulation dose. This balance depends on the NP properties (size, ligand-receptor molecular affinity, and ligand density) and the stage of tumor development (endothelial receptor expression and vascularity) [13].

In this study, we build upon these results to evaluate the tumor response to drug-loaded vascular-bound NPs. As NP accumulation increases within tumor tissue, it follows that the concentration of NP-carried drug reaching the tumor would increase as well; however, treatment efficacy may not strictly depend on drug concentration alone. Accordingly, we develop simulations of vascularized tumor growth to deliver spherical NPs and to track their tumoritropic distribution, accumulation and drug release. We evaluate the change in tumor size as a function of NP properties (size and vascular affinity) and drug properties (loaded amount, release rate, diffusivity). Treatment efficacy is expected to depend on overall cytotoxicity based on these NP and drug parameters.

## Methods

We summarize the computational model as described in [13, 34]. Briefly, the model describes in a 2-D Cartesian coordinate system the viable, hypoxic and necrotic tumor tissue, diffusion of small molecules (cell substrates, oxygen, NPs and drug), and conservation of mass and momentum (as detailed in [34]). The initial condition is a small tumor lesion (<50 μm diameter) in the middle of a pre-existing capillary vasculature grid. Mass conservation equations describe growth (proliferation as a function of total cycling cells) and death from hypoxia (necrosis as a function of oxygen). These equations are combined with diffusion of small molecules to a reaction-diffusion equation. Rate constants for proliferation and apoptosis depend on the availability of cell nutrients and oxygen, and are thus spatiotemporally heterogeneous. The main tumor model parameters are as described in [13]. The transport and progressive accumulation of NPs within the tumor microvasculature are modeled as in [13]. In this study, we extend this model to simulate the release of drug from NPs bound to the vasculature to compare efficacy of treatment based on NP sizes and surface characteristics. The main model equations are summarized in **S1 Appendix**, and the main model parameters are in **S1 Table**.

### Tumor growth

The tumor growth component is based on [35]. Briefly, the tumor tissue is denoted by Ω and its boundary by Σ. In general, tumor tissue may have a proliferating region Ω_*P*_ (typically on the order of 100–200 μm) in which cells have sufficient oxygen and nutrients, a hypoxic region Ω_*H*_ in which oxygen and nutrients are sufficient for survival but not for proliferation, and a necrotic region Ω_*N*_ in which oxygen and nutrients are insufficient for survival. The tumor growth velocity (non-dimensionalized) is implemented via a generalized Darcy’s law [35]:

$$v_{c}=-\mu \nabla P+\chi_{E}\nabla E$$ (1)

where *μ* is cell-mobility representing the net effects of cell-cell and cell-matrix adhesion, *P* is oncotic pressure, χ_*E*_ is haptotaxis, and *E* is ECM density. Definitions for χ_*E*_ and *E* are in [35]. By assuming that the cell density is constant in the proliferating region, the overall tumor growth is associated with the rate of volume change:

$$\nabla \cdot v_{c}=\lambda_{p}$$ (2)

where *λ*_*p*_ is the non-dimensional net proliferation rate (see **Eq (12)** below).

### Angiogenesis

The angiogenesis model component simulates the model by [36] and is based on [34, 35], representing blood flow, vascular leakage and vascular network remodeling due to wall shear stress and mechanical stresses imposed by the tumor tissue. The angiogenesis model is described in detail in [34, 35]. As the tumor grows within the vascular environment, the tissue may experience heterogeneous access to elements diffusing from the vasculature, which may depend on tissue pressure as well as distance from the nearest vascular source.

Briefly, the angiogenesis model assumes that endothelial cells are stimulated to migrate based on chemotaxis due to tumor angiogenic factors (TAF) released by tumor hypoxic tissue and haptotaxis due to gradients of extra-cellular matrix (ECM), as well as random motility. The non-dimensional equation describing the conservation of endothelial cells is [35]:

$$\frac{\partial n}{\partial t}=\nabla \cdot (D\nabla n)-\nabla \cdot \left(\chi_{sprout}^{T}(T)n\nabla T\right)-\nabla \cdot \left(\chi_{sprout}^{E}n\nabla E\right)$$ (3)

where *n* is the non-dimensional endothelial cell density per unit area, and *T* and *E* are the TAF and ECM concentrations, respectively. The diffusion (random migration) coefficient *D* is assumed constant, while the chemotactic and haptotactic migration are described by $\chi_{sprout}^{T}$ and $\chi_{sprout}^{E}$, respectively [35]. The displacement of individual endothelial cells, occurring at the tips of growing sprouts, is given by the discretized form of **Eq (3)** [35]. For the blood flow, an inflow and an outflow pressure are specified as in [34]. As the tumor grows due to cell proliferation, it remodels the surrounding vessels and leads to the creation of new vessels due to a net balance of pro-angiogenic factors secreted by hypoxic tumor cells in the microenvironment.

### Transport of oxygen

The transport of oxygen *σ* through tumor tissue is simulated from the location of extravasation from the vasculature. Oxygen is supplied from the neo- and pre-existing vasculature with extravasation rates $\lambda_{ev}^{\sigma }=\lambda_{neo}^{\sigma }$ and $\lambda_{ev}^{\sigma }=\lambda_{pre}^{\sigma }$, respectively, diffuses with a coefficient *D*_*σ*_, is taken up by both normal cells (with a rate $\lambda_{tissue}^{\sigma }$) and tumor cells ($\lambda_{tumor}^{\sigma }$ in the proliferating region and *q*_*s*_ in the hypoxic region), and decays (with a rate $\lambda_{N}^{\sigma }$) in the necrotic region. Assuming steady-state conditions, the formulation is [15, 34, 35]:

$$0=\nabla •\left(D_{\sigma }\nabla \sigma \right)+\lambda_{ev}^{\sigma }(x,t,1_{vessel},p,\sigma ,h)-\lambda^{\sigma }(\sigma )\sigma$$ (4)

where **x** is position in space, *t* is time, **1**_*vessel*_ is the characteristic function for vasculature (equals 1 at vessel locations and 0 otherwise), *p* is the tumor (solid) pressure, and *h* is the hematocrit in the vascular network which is related to oxygen extravasation (following [35]). The extravasation is modulated by the extravascular interstitial pressure *p*_*i*_ scaled by the effective pressure *p*_e_, with $k_{p_{i}}$ being the weight of the convective transport component of small molecules [15]:

$$\lambda_{ev}^{\sigma }={\bar{\lambda }}_{ev}^{\sigma }1_{vessel}(x,t){\left(\frac{h}{{\bar{H}}_{D}}-{\bar{h}}_{\min}\right)}^{+}\left(1-k_{p_{i}}\frac{p_{i}}{p_{e}}\right)(1-\sigma )$$ (5)

Constants ${\bar{H}}_{D}$ and ${\bar{h}}_{\min}$ represent normal and minimum blood hematocrit required for oxygen extravasation, respectively, and ${\bar{\lambda }}_{ev}^{\sigma }$ is the (constant) transfer rate from both pre-existing and tumor-induced vessels.

### Nanoparticle Accumulation

The vascular accumulation of NPs is modeled as in [13]. Briefly, the accumulation is regulated by the interaction between dislodging hydrodynamic forces and adhesive particle-cell interactions due to formation of ligand-receptor molecular bonds or other non-specific interactions (van der Waals, electrostatic, and steric). The probability of NP adhesion to the vascular endothelium is a function of the NP properties–size, shape, and surface density of ligands–and local vascular biophysical conditions–wall shear rate and surface density of receptors. For spherical NPs, the number *n* of particles with diameter *d* adhering per surface area within a blood vessel with shear rate *S* can be written as [32]:

$$n=n_{0}\alpha d^{\delta_{1}}\;\exp\left[-\beta \left(1+\gamma d^{\delta_{2}}\right)S\right]$$ (6)

where *n*_0_ is the number of NPs per area exposed to the vessel walls and the parameters *α*, *β*, and *γ* are respectively proportional to *i*) the surface density of receptors on endothelial cells (*m*_*r*_) and ligands on the NP (*m*_*l*_), and the ligand-receptor affinity under zero external force (*K*_*A*_^*0*^); *ii*) the characteristic length scale of the ligand-receptor bond (*χ*) and the viscosity of fluid (*μ*); *iii*) the inverse of the surface density of receptors. The coefficients *δ*_1_ (~ 0.45) and *δ*_2_ (~ 1.57) are derived from the best fit of **Eq (6)** with the experimental data shown in [37]. For typical values of *m*_*r*_ = 10^12^ #/m^-2^; *m*_*l*_ = 10^14^ #/m^-2^ and *K*_*A*_^*0*^ = 10^−14^ m^2^, the parameter *α* = *O*(10^12^) m^-2^ [32, 38]. For lower ligand-receptor affinities, *α* is correspondingly lower. For typical values of *m*_*r*_ = 10^12^ #/m^-2^, *χ* = 10^−10^ m^-1^ and *μ* = 10^−3^ Pa s^-1^, the parameter *β* = *O*(10^−4^) m^-2^ s. The parameter *γ* = *O*(10^4^) m^-δ2^. For simplicity, a uniform concentration of NPs in the blood is assumed from the upstream with the maximum normalized to the value of 1.

Multiplying both sides of **Eq (6)** by the surface area of each vessel segment, *S*_*u*_ yields the particle number *N* attached in each vessel segment:

$$N(d,R_{u},S_{u})=S_{u}\alpha d^{\delta_{1}}\;\exp\left(-\beta (1+\gamma d^{\delta_{2}})Srt_{u}\right)$$ (7)

where the shear rate is *Srt*_*u*_ = 4*Q*_*u*_ / *πR*_*u*_^3^ and *Q*_*u*_ is the flow rate. The particle concentrations in the blood (*C*_*p*_) per m^3^ and on the vessel surface (*C*_*pS*_) are solved by the mass conservation equations in the vessels and on their surface, respectively:

$$\left(1+\frac{\Delta t}{V_{p}}\sum_{u}Q_{u})C_{p}^{t+\Delta t}=C_{p}^{t}+\frac{\Delta t}{V_{p}}(\sum_{Qu}C_{u}^{t}Q_{u}(1-N(d,R_{u},S_{u}))\right)$$ (8)

$$C_{pS}^{t+\Delta t}=C_{pS}^{t}+\frac{\Delta t}{S_{p}}\sum_{Q_{u}}C_{u}^{t}Q_{u}N(d,R_{u},S_{u})$$ (9)

where the *u*’s represent the upstream neighbor nodes and *Q*_*u*_’s are the flow rates from nodes *u*’s to node *p*. We summarize the description as in [13]. In **Eq (8)**, the change in particle concentration in the blood depends on what flows in (left- hand side) and what flows out (first term, right-hand side) and what adheres (second term, right-hand side). The change in particle concentration on the surface (**Eq (9)**, left hand side) depends on the amount that flows in (first term, right hand side), plus the amount that adheres (second term, right hand side). The parameters *V*_*p*_ and *S*_*p*_ represent the overall volume and surface area, respectively, for the vessel segment from all the upstream *u*’s to *p*. The concentration *C*_*pS*_ accumulates from *C*_*p*_ over the treatment time (e.g., from the injection time to the time when the NPs in the blood are washed out of the system). The fraction of NPs attached to the surface of each vessel segment is calculated as *M*_*pS*_ = *S*_*p*_ *C*_*pS*_.

### Transport of drug

The drug *G* is released by NPs accumulated in both angiogenesis-induced and pre-existing vessels, and diffuses through the tissue with diffusivity *D*_*G*_. The uptake by tumor and normal cells and the washing away from the interstitial space are included as a combined effect in the rate ${\bar{\lambda }}_{decay}^{G}$, which reflects the drug half-life (assumed here to be 6 hours, similar to paclitaxel for 6-to-24hr infusion):

$$\frac{\partial G}{\partial t}=\nabla \cdot (D_{G}\nabla G)+\lambda_{release}^{G}(t,C_{pS},d)-{\bar{\lambda }}_{decay}^{G}G$$ (10)

We assume that the initial drug loaded is linearly proportional to the NP diameter *d*, and that the release rate ${\bar{\lambda }}_{release}^{G}$ in time is proportional to the square root of this diameter:

$$\lambda_{release}^{G}=kC_{pS}\sqrt{d}$$ (11)

where *k* is a proportionality constant describing the drug release. In case the drug currently loaded is less than what would be released as described in the equation, then the entire loaded drug is released.

As indicated by **Eq (11)**, the release profiles of drug from the accumulated NPs are for simplicity assumed to be linear. The 100 nm NPs were calibrated to fully release their drug load in 12 hours, and the remaining NP groups had release profiles based on the proportionalities discussed previously. Consequently, 1000 nm NPs released drug 3.16 times longer than 100 nm NPs (total of 38 hours vs. 12 hours, respectively).

For all the diffusion equations, as well as the pressure and angiogenic factors, the conditions at the boundaries are $\frac{\partial B}{\partial n}=0$ (zero Neumann condition), where *B* is the element at the boundary (either oxygen, drug, pressure, or angiogenic factors).

### Drug effect on the tumor

Following [16], we assume that the net proliferation rate *λ*_*p*_ (**Eq (2)**) is proportional to the amount of nutrient present. This rate is modulated by the effect of the drug:

$$\lambda_{p}=\{\begin{array}{ll} 0 & \mathrm{outside}\;\Omega \\ \lambda_{\text{M}}\sigma (1-{\bar{\lambda }}_{effect}G)-\lambda_{\text{A}} & \mathrm{in}\;\Omega_{P}\\ 0 & \mathrm{in}\;\Omega_{H}\\ -\lambda_{\text{N}} & \mathrm{in}\;\Omega_{N} \end{array}$$ (12)

In order to simulate the typically cell-cycle dependent effects of chemotherapeutic drugs (as is the case with paclitaxel), the drug is assumed to only act upon proliferating cells. The term ${\bar{\lambda }}_{effect}$ is the rate of drug-induced cell death, *λ*_M_ is the mitosis rate, *λ*_A_ is the apoptosis rate, and *λ*_N_ is the rate of volume loss in the necrotic regions assuming that cellular debris is constantly degraded and the resulting fluid is removed. For simplicity, cell death is assumed to be an instantaneous process. Note that when ${\bar{\lambda }}_{effect}G<1$, the net proliferation is reduced, and when ${\bar{\lambda }}_{effect}G\geq 1$, cell death is introduced and contributes to tumor shrinkage [16].

## Results

### Simulation of Nanoparticle Transport and Coupled Drug Release

We simulate the transport and accumulation of NPs within the tumor vasculature, followed by drug release from the NPs. The systemically injected NPs reach the tumor tissue through both the pre-existing vascular network and the more disorganized neovasculature, which is produced by the tumor through angiogenic stimuli over time. In characterizing tumoritropic NP accumulation, we model spherical NPs spanning a range of sizes: 100, 600 and 1,000 nm. Additionally, we vary the surface density and molecular affinity of the ligand molecules on the NPs surface through variation of the parameter α, as described in **Methods**. First, we assess the vascular accumulation of NPs and compare their relative distribution based on size and surface properties. We then evaluate drug release coupled to the accumulated NPs, and analyze the relative tumor response for each NP group. Finally, we study how variation in drug diffusivity (e.g., as would be expected for different types of drugs) interacts with variation in the NP parameters to affect the tumor response.

### Tumor Development

**Fig 1:**
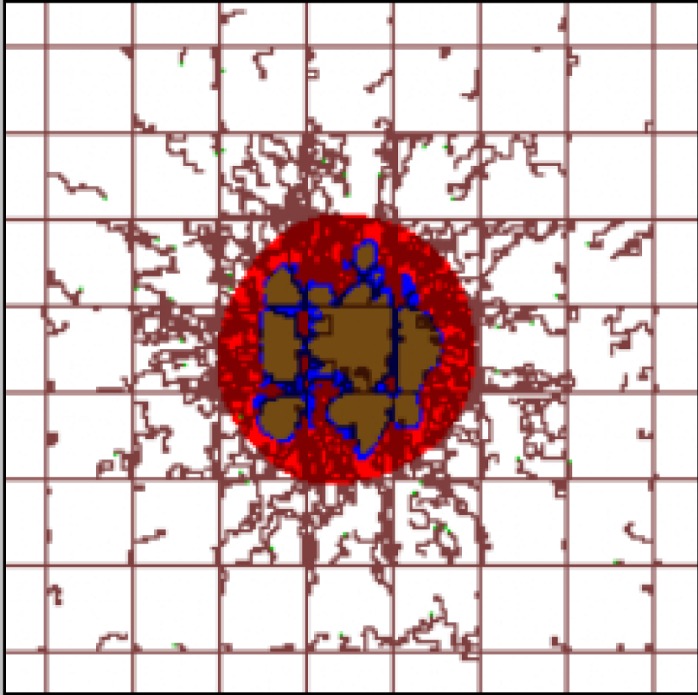
Simulated tumor lesion (~750 μm diameter) right before systemic injection of NPs at day 18 after inception. Three distinct tumor tissue regions, namely, viable, hypoxic, and necrotic, are represented respectively by red, blue, and brown colors. Pre-existing capillary vessels, represented as straight brown lines, are laid out in a regular grid, maintaining normoxic conditions in the surrounding tissue. Angiogenesis-induced capillaries (irregular brown lines) sprout from pre-existing vasculature in response to pro-angiogenic factors released by the hypoxic tissue. Field of view is 2 x 2 mm.

At the moment of inception, an avascular tumor nodule of radius <50 μm is placed in the center of the capillary grid. The nodule grows and develops in time with three identifiable regions: proliferating tissue (red) developing in well-vascularized areas; necrotic tissue (brown) located deep within the tumor away from vasculature; hypoxic tissue (blue) located between the viable and necrotic areas based on the distance from the oxygen-releasing vasculature. **Fig 1** shows the tumor lesion right before systemic injection of NPs at day 18 after inception. The pre-existing vessels are in a regular grid with vessels located every 250 μm along each dimension (brown lines), establishing normoxic conditions to the surrounding tissue, as previously simulated [13, 15, 34]. Irregular vessels (brown) sprout from the pre-existing vessels in response to angiogenic stimuli elicited by hypoxic tissue within the tumor.

### Nanoparticle Uptake and Drug Release

**Fig 2:**
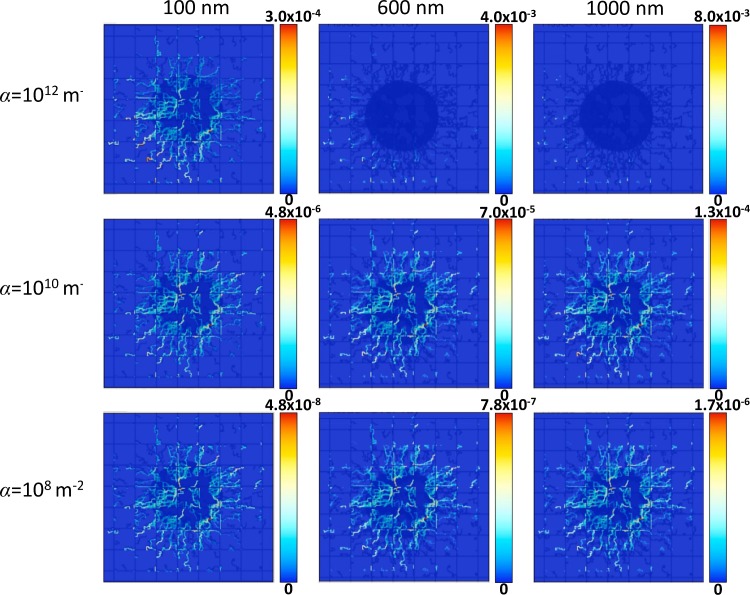
Distribution of vasculature-bound NPs immediately after injection overlaid with the tumor (dark blue shadow) and vasculature (dark blue lines). NP concentration is in dimensionless units, with red indicating highest local value and dark blue indicating lowest. The columns denote different NP sizes, namely 100, 600 and 1,000 nm, while the rows denote different values for the parameter *α* in the tumor-induced neovasculature (*α* = 10^12^ m^-2^ top row; *α* = 10^10^ m^-2^ middle row and *α* = 10^8^ m^-2^ bottom row). For all cases, *β* = 10^−4^ m^-2^ s while *α* for the pre-existing vessels is 100X smaller than for the neovasculature. Field of view for each panel is 2 x 2 mm.

**Fig 2** highlights the trade-off between NP distribution and accumulation as functions of NP vascular affinity and NP diameter. NPs of three different sizes, namely 100 nm (left column), 600 nm (middle column) and 1000 nm (right column), are systemically injected at day 18 to reach tumors having different levels of vascular receptor expressions in the neovasculature, namely α = 10^12^, 10^10^ and 10^8^ m^-2^. In the pre-existing vessels, α is 100 times smaller than in the neovasculature, which is representative of the NP affinity for host tissue-associated vessels. The figure shows how immediately after injection the NPs distribute more uniformly throughout the vasculature as the affinity decreases, but as previously shown [13], the maximum concentration lowers significantly with lower α (about two orders of magnitude lower for each 10^2^ decrease in α). For simplicity, “uniformity” is here defined as the extent by which the NPs spatially overlay with the tumor area, as indicated by their concentration. For the same value of affinity, however, the maximum NP concentration increases with larger size. For the larger 600 and 1000 nm NPs, the NP accumulation in the vasculature can be highly non-uniform. In particular, for α = 10^12^ m^-2^, the NPs accumulate preferentially at the periphery of the tumor (at the vascular inflow side) as a result of high affinity for the neovasculature, which leads to depletion downstream and results in poor distribution throughout the tumor tissue.

**Fig 3:**
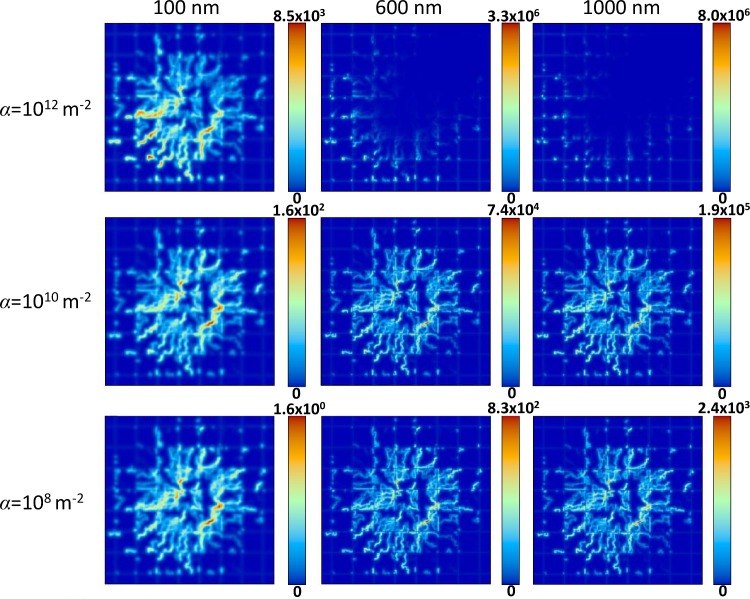
Distribution of drug released by NPs 24 hours after NP injection. Drug concentration is in dimensionless units, with red indicating highest local value and dark blue indicating lowest. Columns and rows denote the same conditions as in Fig 2. Field of view for each panel is 2 x 2 mm.

The distribution and accumulation of NPs directly influences the corresponding drug distribution. **Fig 3** shows that drug release from the accumulated NPs (**Fig 2**) into the surrounding tissue follows similar concentration trends as observed for the NP distribution, with the maximum drug concentration in this case decreasing about an order of magnitude for each 10^2^ decrease in α. For all cases, *D*_*G*_ = 0.022 in **Eq (10)**, representing a “standard” value for drug diffusivity. For the same value of vascular affinity, the maximum drug concentration increases with NP size following the drug release rate specified in **Methods**.

**Fig 4:**
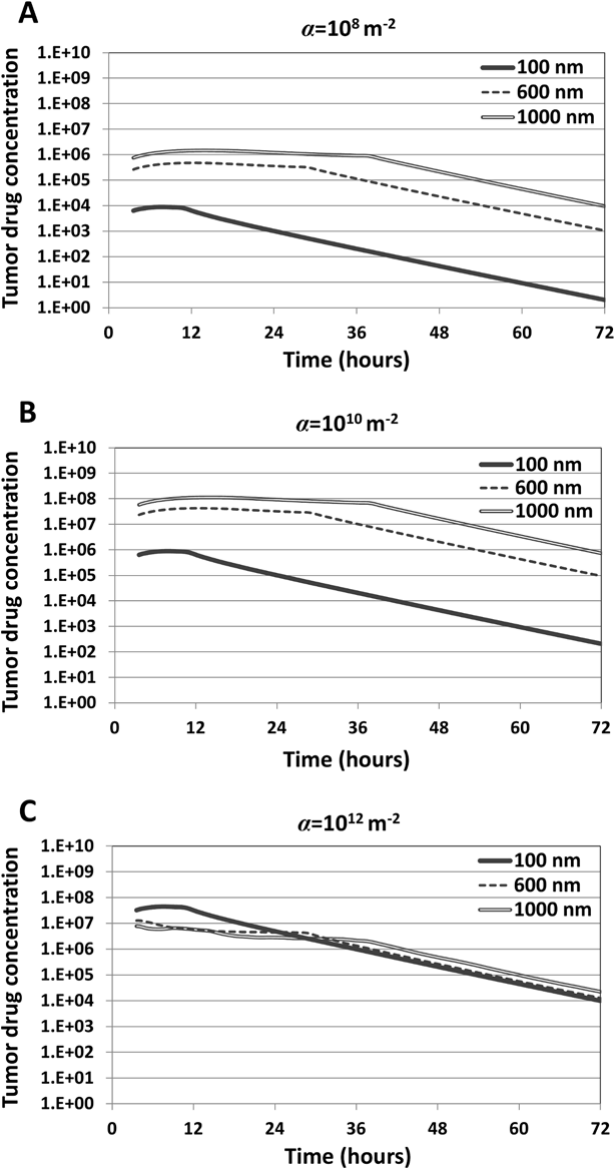
Drug concentration within the tumor tissue attained by accumulated NPs(dimensionless units) as a function of NP size and vascular affinity (parameter *α*, with values shown for the tumor-induced neovasculature). The peaks for each curve correspond to the time when the NPs have fully released their drug. The drug concentration is determined by the cellular uptake and decay rates, as described by Eq (10) in Methods.

Next, we evaluated drug release rates and total drug release proportional to NP size as described by **Eq (11)**. The fraction of drug localized in the tumor tissue is shown in **Fig 4**. Although the NPs simulated do not correspond to a specific formulation, it is reasonable to assume that as NP diameter increases, the total amount of drug loaded increases as well due to a higher internal volume. Additionally, drug release rates are assumed to increase with increasing NP size due to a greater surface area for drug to diffuse out. As a result of the parameter selection described in **Methods**, the larger NPs will release drug at a higher rate, and a higher drug loading allows the larger NPs to have a correspondingly longer sustained release. The peaks for each curve highlight the time at which the NPs have fully released their drug payload. As the affinity of the NPs for the vasculature increases (**Fig 4A** and **4B**), the released drug concentration correspondingly increases for all NP sizes.

Despite most drug being released by the larger NPs, **Fig 4** shows that the drug concentration within tumor tissue is dependent on the vascular affinity of the NPs and not solely determined by NP size. For the lower affinity cases (*α* = 10^8^ m^-2^ or *α* = 10^10^ m^-2^), larger diameter NPs accumulate at higher concentrations overall and at higher fraction in the tumor (**Fig 2**). Combining this fact with the higher drug loading of the larger NPs, in this case the larger diameter NPs deliver higher drug concentrations to the tumor tissue (**Fig 4A** and **4B**). Interestingly, for the highest affinity case (α = 10^12^ m^-2^), the smallest NPs (100 nm) within the first 24 hours elicit the highest concentrations of drug within the tumor (**Fig 4C**) despite having the lowest drug loading as well as the lowest total accumulation compared to the larger NPs with same affinity (**Fig 2**).

### Analysis of Treatment Efficacy

**Fig 5:**
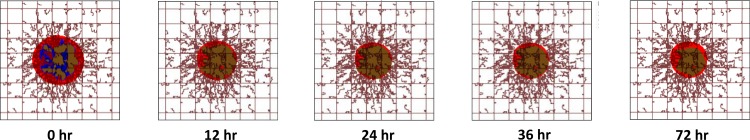
Representative tumor lesion regression due to simulated treatment as a function of time. Based on the hypothetical loading of drug in the NPs for the case of parameter *α* = 10^12^ and diameter *d* = 100 nm, this treatment yielded a 50% reduction in tumor mass by 24 hr. After this time, the lesion begins to regrow as the remaining drug is insufficient to arrest its proliferation, leading to proportionally increased proliferating tissue (red) by 72 hr. Colors and field of view are as in Fig 1.

As described in **Methods**, the drug is assumed to affect the net proliferation rate of viable non-hypoxic tissue only. Low concentrations of drug will slow the net proliferation rate, while higher drug concentrations will cause the proliferating tissue to die at a rate proportional to this concentration. A representative simulation (with parameters α = 10^12^ m^-2^; 100 nm) demonstrating the effect of drug exposure on a tumor mass is shown in **Fig 5**. Over the course of the first 24 hr, the tumor shrinks as the drug is delivered from accumulated NPs. There is a net reduction in the proliferating cell fraction (red) and an increased fraction of necrotic tissue (brown). The hypoxic tissue (blue), immune to the cell-cycling action of the drug, becomes proliferative as access to vasculature is gained following the reduction in overall proliferative fraction. Beyond 24 hr, the tumor begins to recover from the treatment as the drug concentration has decayed low enough for the proliferation rate to overcome the death effect of the drug, leading to proportionally increased proliferating tissue by 72 hr.

As shown in **Fig 3**, each variation of the NP affinity and diameter parameters results in varying maximum drug concentrations and overall drug distribution in the continuum. These variations ultimately alter the net effect of the drug on the tumor proliferation. In order to make a standardized comparison between the treatment efficacy of the different formulations, we first simulated treatment with NPs of each formulation using *D*_*G*_ = 0.022 in **Eq (10)** as a “standard” value for drug diffusivity. Next, we iteratively tuned the parameter ${\bar{\lambda }}_{effect}$, the rate of drug-induced death in **Eq (12)**, until determining the value of ${\bar{\lambda }}_{effect}$ capable of reducing the tumor by 50% of its original area (${\bar{\lambda }}_{IC50}$). Finally, we noted 1/${\bar{\lambda }}_{IC50}$ to be proportional to the overall efficacy of the treatment for each specific NP formulation: if ${\bar{\lambda }}_{IC50}$ is increased, the modeled drug would correspondingly require an increase in the death rate to reach 50% reduction in area.

**Fig 6:**
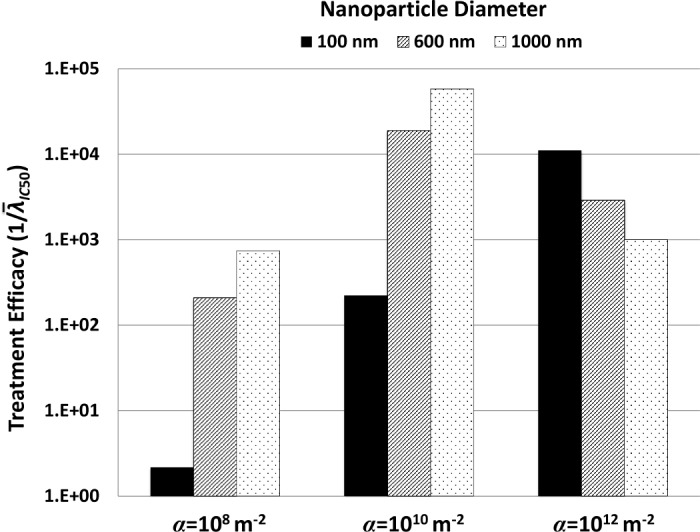
Relative efficacy of all treatments types (three levels of parameter *α*, three levels of NP diameter *d*). Relative efficacy (non-dimensional value) is measured as 1/${\bar{\lambda }}_{IC50}$, with ${\bar{\lambda }}_{IC50}$ being the rate of drug-induced death necessary to reduce the tumor area to 50% of its original size. A larger value of 1/${\bar{\lambda }}_{IC50}$ thus denotes more effective treatment.

The values of 1/${\bar{\lambda }}_{IC50}$ for each NP formulation are presented in **Fig 6**. For lowest and medium affinities (α = 10^8^ and α = 10^10^, respectively), larger NP diameters generally led to increased efficacy. The smallest diameter (100 nm) performed best at the highest affinity (reflecting the result in **Fig 4F**), while the two larger diameters (600 and 1000 nm) were most efficacious with the medium affinity. Regardless of NP size, treatment efficacy was better for medium than for lowest affinity; however, this trend was reversed for highest affinity. In this case, an increased NP diameter decreased the treatment efficacy, due to lower uptake of the larger NPs. At the lowest affinity (α = 10^8^) all NPs were less effective than at higher affinities. Although this case was shown to have more uniform NP distributions (**Fig 2**), a low vascular affinity reduced the overall accumulated NP concentration and associated drug release compared to the other formulations (**Fig 3**).

### Variation in Drug Diffusivity

**Fig 7:**
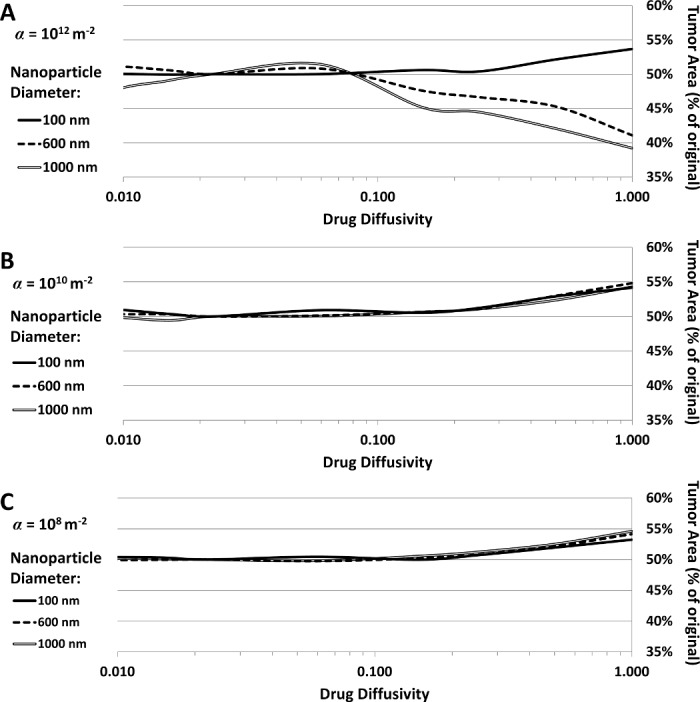
Tumor response to variation in drug diffusivity (*D*_*G*_ in Eq (10)). Tumor area (as % of original) as a function of drug diffusivity for (A) *α* = 10^12^, (B) *α* = 10^10^, and (C) *α* = 10^8^.

Using ${\bar{\lambda }}_{effect}={\bar{\lambda }}_{IC50}$ for each case of NP diameter and affinity, i.e., simulating a 50% reduction in tumor size, we varied the drug diffusivity *D*_*G*_ in **Eq (10)** to evaluate how treatment efficacy would be affected by drugs with differing properties. The values chosen varied from hardly diffusive (*D*_*G*_ = 0.010) to optimally diffusive (*D*_*G*_ = 1, similar to O_2_). The results are presented in **Fig 7**. For the NPs with low or medium affinity (α = 10^8^ or α = 10^10^, respectively), high drug diffusivity seems to slightly decrease treatment efficacy; otherwise, variation in diffusivity does not seem to affect the tumor response. With high affinity (α = 10^12^), the same situation holds for the case of small NPs (100 nm). In contrast, medium- and larger-sized (600 and 1000 nm) NPs with high affinity elicit increasingly higher efficacy for increased drug diffusivity.

## Discussion

In our previous work, a two-dimensional model for the growth of a vascularized tumor was integrated with a mesoscale formulation for the vascular adhesion of systemically injected NPs [13], showing that a nonlinear relationship exists between NP size and affinity to achieve homogeneous intra-tumoral distribution. The tumor formulation included viable, hypoxic, and necrotic tissue representations coupled with an angiogenesis model stimulated by the tumor growth and with blood flow influenced by this growth. In this study, we extend this system to study the tumor response as a function of drug loading and release from vasculature-bound NPs while accounting for inherent variations in drug loading and release from differently sized NPs.

The results show that although larger (600 and 1000 nm) diameter NPs with low or medium vascular affinity are expected to deliver higher drug concentrations within tumor tissue (**Fig 4A** and **4B**), at high affinity they are outperformed by small (100 nm) NPs (**Fig 4C**). In this case, the smallest NPs elicit higher concentrations of drug within the tumor overall despite having the lowest drug loading as well as the lowest total accumulation compared to the larger NPs with same affinity (**Fig 2**). This highlights the benefit of a more uniform NP distribution, even at lower concentrations than with larger NPs that are non-uniformly distributed.

The simulations of treatment efficacy (**Fig 6**) show the complex interplay between NP uptake as a result of size and vascular affinity (**Fig 2**) and drug released within tumor tissue (**Figs 3** and **4**). Efficacy was hampered by low affinity regardless of NP size, as insufficient numbers of NPs were able to accumulate in the tumor vasculature. Larger NP size coupled with low affinity in addition impedes efficacy due to non-uniform NP distribution and drug release (**Figs 3** and **4**). Although higher affinity correspondingly increases efficacy for all NP sizes, the results show that a small NP size (100 nm) would elicit the most tumor regression at highest affinity compared to the larger sizes. The results further show that medium or larger sized NPs coupled with medium affinity would also be expected to achieve high tumor regression (**Fig 6**), due to a more homogeneous NP distribution coupled with higher drug loading and release. While the relationship between affinity and efficacy is linear for the smallest NPs for the range of affinity studied, it is also expected to be nonlinear at even higher affinities, similar to what is shown by the larger NPs.

Interestingly, it has been observed that low-avidity NPs exhibited several-fold higher selectivity of targeting to pathological endothelium compared to high-avidity NPs due to multivalent interactions between NPs and a high density target expressed in pulmonary inflammation [39]. It has also been shown that NP depletion effects at high adhesion efficiency can limit adhesion under fluid flow (e.g., [40]), which lends support for a strategy aiming for submaximal adhesion efficiency.

The results essentially highlight that tumor regression as a function of drug strength (${\bar{\lambda }}_{effect}$ in **Eq (12)**) exhibits a nonlinear relationship dependent on NP affinity and size. These findings are consistent with our previous study [13]. Treatment efficacy requires a balance between NP vascular affinity and NP size in order to achieve a high drug concentration in the tumor; delivering larger NPs with more drug may not necessarily be the best strategy to achieve the most tumor regression. A prolonged bioavailability coupled with uniform distribution would be more effective. This is supported by the study of variation in drug diffusivity (**Fig 7**). One would assume that a more diffusive drug would be capable of reaching more tissue and therefore have more therapeutic benefit; however, the results show that increased drug diffusivity only benefits the heterogeneously distributed cases while negatively affecting the homogeneously distributed NPs. Increased diffusivity allows drug heterogeneously localized to diffuse further through the tumor and thus become more uniform, while increased diffusivity for drug already uniformly distributed leads to dispersion and thus lower cytotoxicity. In practical terms, this means that the drug loaded into the NPs should be selected so that its intrinsic properties, such as molecular size and charge, all of which can affect its diffusivity, would optimize the tumor response based on the expected NP distribution.

In future work, we plan to evaluate specific NP formulations targeted to vascular endothelium coupled with existing chemotherapeutic drugs. This will require expanding the analysis to evaluate other values of the NP parameters, including vascular affinity and size. Moreover, the EPR effect should be taken into consideration for smaller particles. Longer term, calibration of the tumor and vascular parameters to patient-specific tumor data obtained from biopsy or imaging would permit evaluation of potential tumor response to achieve optimal regression, especially for metastatic lesions. In this manner, the design of nanotherapy parameters (including dosing schedules) could benefit from a theoretical framework integrating mathematical modeling and computational simulation with laboratory observation and measurements.

## Supporting Information

S1 Appendix Main equations and parameters related to the computational model of tumor response to drug release from vasculature-bound nanoparticles. (PDF)

S1 Table Main model parameters and associated values. (PDF)
