# Supplementary material for: Computational Modeling of Tumor Response to Drug Release from Vasculature-Bound Nanoparticles
Source: PLoS One. 2015 Dec 14;10(12):e0144888. doi: 10.1371/journal.pone.0144888 (PMC4682796; doi:10.1371/journal.pone.0144888)
Supplement: S1 Appendix — (PDF) [file pone.0144888.s001.pdf]

Tumor Growth

$$V_{boundary} = \mathbf{v}_c \cdot \mathbf{n}$$

Evolution of tumor ($\mathbf{n}$ denotes boundary normal direction)

$$\mathbf{v}_c = -\mu \nabla P + \chi_E \nabla E$$

Non-dimensional tumor velocity $\mathbf{v}_c$ (**Eq. 1**; Ref. 35, Eq. 7)

$$\nabla \cdot \mathbf{v}_c = \lambda_p$$

Growth of tumor (**Eq. 2**; Ref. 35, Eq. 8)

$$\lambda_p = \lambda_{\mathrm{M}}\sigma - \lambda_{\mathrm{A}} \text{ in } \Omega_P \quad \text{and} \quad \lambda_p = \lambda_{\mathrm{N}} \text{ in } \Omega_N$$

Proliferation rate λ_p (**Eq. 12**; Ref. 16. Eq. 13)

$$0 = \nabla \cdot \left(D_\sigma \nabla \sigma\right) + \lambda_{ev}^{\sigma}(\mathbf{x}, t, \mathbf{1}_{vessel}, p, \sigma, h) - \lambda^{\sigma}(\sigma)\sigma$$

Transport of oxygen σ (**Eq. 4**; Ref. 34, Eq. 1)

$$\lambda_{ev}^{\sigma} = \overline{\lambda}_{ev}^{\sigma} \mathbf{1}_{vessel}(\mathbf{x}, t)\left(\frac{h}{\overline{H}_D} - \overline{h}_{\min}\right)^{+}\left(1 - k_{p_i}\frac{p_i}{p_e}\right)(1-\sigma)$$

Oxygen extravasation rate λ_{ev}^{σ} (**Eq. 5**; Ref. 34, Eq. 3)

Angiogenesis

$$\frac{\partial n}{\partial t} = \nabla \cdot (D \nabla n) - \nabla \cdot \left(\chi_{\text{sprout}}^{T}(T) n \nabla T\right) - \nabla \cdot \left(\chi_{\text{sprout}}^{E} n \nabla E\right)$$

Endothelial cell density n (**Eq. 3**; Ref. 35, Eq. 21)
(discrete and stochastic implementation)

$$0 = \nabla \cdot (D_T \nabla T) + \overline{\lambda}_{\text{prod.}}^{T}(1-T)\mathbf{1}_{\Omega_H} - \overline{\lambda}_{\text{decay}}^{T} T - \overline{\lambda}_{\text{binding}}^{T} \mathbf{1}_{\text{sprout tips}}$$

TAF concentration T (Ref. 35, Eq. 20)

$$\frac{\partial E}{\partial t} = -\overline{\lambda}_{\text{degradation}}^{E} EM + \overline{\lambda}_{\text{prod.}}^{E}(1-E)\mathbf{1}_{\Omega_V} + \overline{\lambda}_{\text{sprout prod.}}^{E} \mathbf{1}_{\text{sprout tips}}$$

ECM density E (Ref. 35, Eq. 18)

$$\frac{\partial M}{\partial t} = \nabla \cdot (D_M \nabla M) + \overline{\lambda}_{\text{prod.}}^{M}(1-M)\mathbf{1}_{\Omega_V} - \overline{\lambda}_{\text{decay}}^{M} M + \overline{\lambda}_{\text{sprout prod.}}^{M} \mathbf{1}_{\text{sprout tips}}$$

MDE concentration M (Ref. 35, Eq. 17)

Blood Flow

$$\sum_q Q_{qp} = O_V$$

Flow conservation for each vascular node (Ref. 34, Eq. 22)

$$O_V = K_{Vf} S_V (P_c - P_f)$$

Fluid extravasation at a node (Ref. 34, Eq. 23)

$$\Delta R = (S_{wss} + S_p + S_m - S_s) R \Delta t$$

Vessel radius R adaptation (Ref. 34, Eq. 25)

Nanoparticles

$$N(d, R_u, S_u) = S_u \alpha d^{\delta_1} \exp\left(-\beta(1 + \gamma d^{\delta_2}) Srt_u\right)$$

Particle number N attached in a vessel segment (**Eq. 7**; Ref. 13, Eq. 14)

$$Srt_u = 4Q_u / \pi R_u^{3}$$

Shear rate Srt in vessel segment u (Ref. 13)

$$\left(1 + \frac{\Delta t}{V_p}\sum_u Q_u\right) C_p^{t+\Delta t} = C_p^{t} + \frac{\Delta t}{V_p}\left(\sum_{Qu} C_u^{t} Q_u \left(1 - N(d, R_u, S_u)\right)\right)$$

Particle concentration C_p in blood (**Eq. 8** ; Ref. 13, Eq. 15)

$$C_{pS}^{t+\Delta t} = C_{pS}^{t} + \frac{\Delta t}{S_p}\sum_{Q_u} C_u^{t} Q_u N(d, R_u, S_u)$$

Particle concentration C_{pS} on vessel surface (**Eq. 9** ; Ref. 13, Eq. 16)

Drug

$$\frac{\partial G}{\partial t} = \nabla \cdot (D_G \nabla G) + \lambda_{release}^{G}(t, C_{pS}, d) - \overline{\lambda}_{decay}^{G} G$$

Drug concentration G (**Eq. 10**)

$$\lambda_{release}^{G} = k C_{pS}\sqrt{d}$$

Drug release rate $\lambda_{release}^{G}$ (**Eq. 11**)

$$\lambda_p = \lambda_{\mathrm{M}}\sigma(1 - \overline{\lambda}_{effect} G) - \lambda_{\mathrm{A}}$$

Effect of drug on tumor net proliferation λ_p (**Eq. 12**; Ref. 16, Eq. 13)
