# Supplementary material for: Computational Modeling of Tumor Response to Drug Release from Vasculature-Bound Nanoparticles
Source: PLoS One. 2015 Dec 14;10(12):e0144888. doi: 10.1371/journal.pone.0144888 (PMC4682796; doi:10.1371/journal.pone.0144888)
Supplement: S1 Table — (PDF) [file pone.0144888.s002.pdf]

S1 Table. Main model parameters and associated values. All other model parameters are as in [1].
(*) Value is rescaled by the square of the simulation system characteristic length (1 cm) and divided by the system characteristic time (1 sec) multiplied by the oxygen diffusivity [2] (1 x 10^{-5} cm^2 s^{-1}).

**Parameter**	**Value**	**Reference**
Tumor proliferation rate	1 day^{-1}	[3]
Tumor necrosis threshold	0.5700	[3]
Tumor hypoxic threshold	0.5750	[3]
Oxygen diffusivity	1 (*)	[4]
Oxygen transfer rate from vasculature	5 (*)	[4]
Oxygen uptake rate by proliferating tumor cells	1.5 (*)	[4]
Oxygen uptake rate by hypoxic tumor cells	1.3 (*)	[4]
Oxygen uptake rate by tumor microenvironment	0.12 (*)	[4]
Oxygen decay rate	0.35 (*)	[4]
NP diameter d	100; 600; 1,000 nm	[5]
NP drug load	(variable)	Proportional to d
NP drug release	(variable)	Proportional to $\sqrt{d}$
Drug diffusivity	0.6 (*)	Initial value; varied over [0.01,1.00]
Drug decay rate	6hr half-life	Similar to Paclitaxel

References:

1. Wu, M., Frieboes, H.B., McDougall, S.R., Chaplain, M.A., Cristini, V., Lowengrub, J. The effect of interstitial pressure on tumor growth: Coupling with the blood and lymphatic vascular systems. J Theor Biol 320, 131-151 (2013).
2. Nugent, L.J., Jain, R.K. Extravascular diffusion in normal and neoplastic tissues. Cancer Res 44, 238-244 (1984).
3. van de Ven, A.L., Wu, M., Lowengrub, J., McDougall, S.R., Chaplain, M.A., Cristini, V., et al. Integrated intravital microscopy and mathematical modeling to optimize nanotherapeutics delivery to tumors. AIP Adv 2, 11208 (2012).
4. Macklin, P., McDougall, S., Anderson, A.R., Chaplain, M.A., Cristini, V., Lowengrub, J. Multiscale modelling and nonlinear simulation of vascular tumour growth. J Math Biol 58, 765-798 (2009).
5. Frieboes, H.B., Wu, M., Lowengrub, J., Decuzzi, P., Cristini, V. A computational model for predicting nanoparticle accumulation in tumor vasculature. PLoS One 8, e56876 (2013).
